# Spaces of Exclusion and Neglect: The Impact of COVID-19 on People With Disabilities in Bangladesh, Kenya, Nepal, Nigeria, and Uganda

**DOI:** 10.1177/12063312231159208

**Published:** 2023-03-06

**Authors:** Stephen Thompson, Brigitte Rohwerder

**Affiliations:** 1 Institute of Development Studies, University of Sussex, Brighton, UK

**Keywords:** COVID-19, disability inclusion, Bangladesh, Kenya, Nepal, Nigeria, Uganda

## Abstract

This research investigates how COVID-19 has affected experiences of people with disabilities in low- and middle-income contexts. A qualitative approach was used to collect data as the pandemic progressed from 75 participants in Nigeria, Bangladesh, Nepal, Kenya, and Uganda. The research aimed to be inclusive of people with disabilities by asking the participants directly about their perspectives with a narrative interview method being employed to gain each person’s unique insights. A participatory thematic analysis of the data, followed by a spatial analysis process, produced rich and highly individualized accounts of the spatiocultural experiences relating to how people with disabilities had occupied both private and public space during the pandemic. Differing factors, such as the dominant culture, gender, a person’s impairments, and the social environment, are shown to shape people’s experiences. Across cultures, COVID-19 is shown to have presented new challenges for people with disabilities while preexisting disadvantages have been exacerbated.

## Introduction

When the scale and severity of the COVID-19 pandemic became apparent, many organizations predicted that the situation could result in exacerbated marginalization for people with disabilities (International Disability Alliance [IDA], 2020; International Disability and Development Consortium [IDDC] & IDA, 2020; Office of the United Nations High Commissioner for Human Rights [OHCHR], 2020; World Health Organization [WHO], 2020). Concern was raised with regard to how the pandemic might affect how people with disabilities might access health care services, water and sanitation services, education, and public health information. Without appropriate action, people with disability were also believed to be at greater risk of contracting COVID-19 for a variety of reasons, including barriers to hygiene, difficulty in enacting social distancing, and barriers to accessing public health information. In addition, depending on individual health conditions, infection may be more severe when it occurs. Disruptions to support services were also predicted to result in people with disabilities experiencing disproportionate impact of the pandemic (WHO, 2020). Warnings were issued that the COVID-19 response may result in people with disabilities being disproportionately affected due to attitudinal, environmental, and institutional barriers being reproduced. People living in institutions were highlighted as being particularly at risk (OHCHR, 2020). A lack of participation of people with disabilities in designing and implementing protective measures was highlighted as an area for particular concern (IDDC & IDA, 2020). Intersecting inequalities was also a concern with recognition that women with disabilities may be particularly marginalized as the crisis develops (IDA, 2020).

As the pandemic progressed, research began to show how these concerns had come to pass in a variety of settings and contexts. The COVID-19 crisis was shown to exacerbate existing challenges experienced by people with disabilities, as well as creating some new ones (Cahapay, 2021; Goyal et al., 2020; Huong, 2020). The extensive literature review by Kubenz and Kiwan (2021) documented the impact of the pandemic on people with disabilities in low- and middle-income countries. It shows that in COVID-19 times, people with disabilities faced increased discrimination across many sectors of society, including health, education, and work. The risk of violence and poverty also increased for this group (Kubenz & Kiwan, 2021).

To gain a better understanding of the impact of COVID-19 on people with disabilities, how disability is conceptualized must be clear. Following the social model, disability is understood to be constructed by society. Disability is regarded as structural, whereas, in contrast, impairment is regarded as individual. By defining disability as a societal creation, the social model differentiates itself from the medical or individual model that inherently focuses on a deficit or problem relating to an individual (Shakespeare, 2010). Barnes (2012) describes the social model of disability as a tool that can be used to better understand the disabling tendencies of society. Perspectives relating to the social model can inform policies and practices to address such situations. He argues that while impairment may be a constant, disability does not need to be and should not be. The societies we live in have the potential to realize positive change resulting in a more inclusive world. Society decides how accommodating and inclusive it is or is not. As such, the context and culture relating to where someone with impairments lives becomes important to understanding how they experience disability.

National responses to COVID-19 have varied in terms of approach, severity, and the length of time restrictions have lasted for. Many responses involved social distancing, lockdowns of all public life, and quarantine.

In Bangladesh, to combat COVID-19, the government took a hard-line approach canceling all public celebrations and banning gatherings. In addition, all government and private offices were closed, as were schools. Transportation services were shut down, including domestic and international flights. Law enforcement agencies were deployed to enforce strict social distancing (Islam et al., 2020). Nigeria’s response was not as severe, but also included travel restrictions and social distancing. The Nigerian COVID-19 measures also introduced contact tracing, self-isolation, and quarantine (Jacobs & Okeke, 2022). In Nepal, the government introduced lockdowns, border closing, and travel restrictions, but efforts were undermined by a surge in both internal migration and workers returning from overseas (Thapa, 2021). In Kenya, schools were closed, and workers were advised to work from home where possible. All mass gatherings were prohibited. In addition, a nationwide dawn to dusk curfew was introduced, but movement restrictions were mainly focused on urban areas with high COVID-19 transmission rates (Wangari et al., 2021). Uganda’s approach was also more targeted, including restricting travel to and from high-risk countries and mandatory institutional quarantine of all incoming travelers. Schools and colleges were closed, as were religious, political, or social gatherings (Sarki et al., 2020). As restrictions varied from country to country, the different context and cultures have also influenced how people with different impairments experienced space during the COVID-19 crisis.

To investigate this further, we undertook some qualitative research as part of the UK Aid-funded Inclusive Futures initiative. This consortium program of 16 global partners, including the Institute of Development Studies (IDS) and led by the non-government organization Sightsavers, focuses on advancing the inclusion of people with disabilities in low- and middle-income settings. The initiative approach involves pooling the collective expertise of the partners and working with Organizations of People with Disabilities (OPDs) to create innovative solutions to improve full and effective participation in society of people with disabilities (Sightsavers, 2021). Our research set out to give people with disabilities in a range of countries the opportunity to describe in their own words how COVID-19 and associated national responses had affected their lives, including impact on their spatial and cultural practices.

Drawing on existing networks and focusing on locations where Inclusive Futures is operating, the research was undertaken in two Asian countries (Nepal and Bangladesh) and three African countries (Nigeria, Kenya, and Uganda). All five countries are signatories to the United Nations Convention on the Rights of Persons with Disabilities (UNCRPD), Article 11 of which details how signature countries are obliged under international law, to take all necessary measures to ensure the protection and safety of persons with disabilities in situations of risk, including in humanitarian emergencies (UN, 2006). In addition to international law, all five countries have national laws and policies focused specifically on the inclusion of people with disabilities. These laws and policies are explored in detail in the literature (Rohwerder, 2020a, 2020b, 2020c; Thompson, 2020a, 2020b). Despite the high-level political rhetoric regarding disability inclusion and commitments to address discrimination, we wanted to hear from people at the grassroots level about their lives and experiences.

The research aimed to expedite full and effective participation and inclusion by asking the participants directly about their perspectives on the impact of the crisis on their lives and the environments they inhabit. The aim was to produce individualized accounts of how people had experienced and occupied space during the pandemic in the five countries. We also sought to hear from the participants on their views about the longer-term consequences of the pandemic and what their post-COVID lives might be like.

## Method

Qualitative methods were selected to focus on the lived experience of participants from Bangladesh, Kenya, Nepal, Nigeria, and Uganda. The research aimed to produce data that honored participants’ reflections and local meanings (Hammett et al., 2015; Tracy, 2012). Similar qualitative methods have been shown to produce data that can “provide insight into cultural activities that might otherwise be missed in structured surveys or experiments” (Tracy, 2012, p. 29). The research was designed with the UNCRPD general principles in mind, with specific attention paid to respect for inherent dignity, individual autonomy, nondiscrimination, full and effective participation and inclusion, respect for difference, equality of opportunity, accessibility, and equality between men and women (UN, 2006).

With this framing in mind, narrative interviews were selected as a method. Narrative interviews are a form of unstructured, in-depth interview based on story-telling and listening. They offer a participant-led alternative to the question–response schema of traditional interviews that rely on questions followed by responses that are prone to researcher-imposed structure, language, and focus. The structure and operation of the narrative interview should be deliberately arranged to minimize the interviewer’s influence (Thompson et al., 2021). The COVID-19 pandemic and national responses have resulted in an unprecedented global situation for the vast majority of the global population. Experiences have been shaped by context. Narrative interviews allow for an exploration of these experiences by inviting participants to tell their story. This approach was further deemed appropriate as it has been shown to be suitable across contexts as storytelling is found across cultures and space. Also, narrative interviews can be successfully undertaken regardless of participant status with regard to various factors, including education and communication competence, which is important given the focus of the research (Jovchelovitch & Bauer, 2000). Narrative is described by Frank (2017) as the means through which people understand and express the subjective, value-laden, and a-rational experience of real life. Narratives thus allow people to express in their own terms what it means to be living. As a “teller-focused” approach, narrative interviews allow participants to talk about various sensitive types of human experience, including those that may be complex or difficult to bring up (Hydén, 2014).

In terms of background and positionality, the authors are European and work at a U.K.-based university. Neither author has a disability themselves. To ensure that the social and cultural contexts of the five countries were considered during the research process, seven local researchers were employed, allowing the interviews to be undertaken in the participant’s chosen language (Swahili, Luganda, Nepali, Bengali, or English). Three of the researchers identified as being disabled themselves. Five of the researchers (including one researcher with disabilities) had experience of participatory research. Two of the researchers with disabilities were relatively inexperienced and were provided with training and mentoring to develop their research skills and capacity. The local researchers were regarded as more than just data collectors, as they engaged in the analysis and validation of the research. Further details on the research team are detailed in Wickenden et al. (2021). Ethics approval was obtained from the IDS ethics committee.

The Inclusive Futures consortium partners worked with partner OPDs to identify participants who had been involved as beneficiaries in program initiatives to address stigma and discrimination, improve access to health, and make education and work inclusive for people with disabilities. Recruitment was purposive to ensure balance with regard to gender, age, geographical location, and impairment types. Participants with intellectual impairments, and multiple and complex disabilities (such as deafblindness) were purposively included, as these people are often unfairly excluded from research undertaken in various contexts (Shepherd et al., 2019; Skilton et al., 2018). All participants were over 18 years old. Two participants were parents of children with disabilities.

The research was undertaken during the COVID-19 pandemic in 2020. To overcome ethical risks relating to the safety of both participant and researcher, the interviews were conducted virtually either through video messaging or on the phone. Video calls were encouraged, to allow a greater connection between interviewer and interviewee. Participants were reimbursed for data usage. Undertaking narrative interviews remotely has been shown in other contexts to be appropriate and in certain circumstances a preferred alternative to face-to-face interviews (Holt, 2010). Technical assistance and personal support, including sign language or tactile interpreters and/or other assistance, were provided to ensure participants could connect with the interviewers and had their access needs met.

After an initial call to introduce the research purpose and process (including consent, the right to withdraw, and anonymization of data) and confirm accessibility needs, each participant was invited to be interviewed twice, between 1 and 2 months apart. The second interview provided the opportunity for participants to follow up with experiences they had forgotten to mention or had not felt confident enough to express. In addition, it allowed for an understanding to be developed of how experiences had changed over time, especially as the second interview took place at a time when restrictions were generally easing. This was particularly important as the progression of the pandemic was experienced differently in the five focus countries, with national responses being contextual and varied.

During the narrative interview, one very general and open question was asked, allowing the participant to direct the response how they chose (Jovchelovitch & Bauer, 2000). The question was, “How has the COVID-19 situation developed for you and impacted on your life?” The researchers could use prompts and follow-up questions to gain more information on key points that participants chose to talk about. After each interview call, a follow-up call was made to check on the well-being of the participant, recognizing that talking about the COVID-19 pandemic could trigger strong emotions.

The interview calls lasted up to an hour. The researchers made notes during the interview and took audio recordings, which were then translated and transcribed. The researchers also kept reflexive diaries and provided summaries of their reflections to the wider team (Dowling et al., 2016). After the two rounds of data collection, collective analysis sessions with the IDS and in-country researchers were held, involving thematic analysis (Braun & Clarke, 2012). The collective generation of themes ensured that the process was grounded with perspectives from researchers from the five countries in focus.

Online participant validation events were held once the initial analysis had been completed. These events took the form of participatory workshops and were inspired by the ideas of Chambers (2002), whose work focuses on participatory learning and change, aiming to include people who are often voiceless, marginalized, or left behind. They were made possible through the support of the partner OPDs, who advised and assisted with various considerations relating to access and communication needs of participants. As well as offering sign-language interpreters as needed, participants were offered the opportunity to be brought to the OPD offices, where support could be provided. This resulted in some participants joining the events as a group. Others opted to join individually from home or work. Some had assistance from family members or friends to participate. To ensure the events were as accessible as possible, extra time was factored in to ensure that everyone who wanted to contribute had the chance to do so. The cost of the data to join the meetings was reimbursed to ensure that this was not a barrier to participation. Slides and other visuals were kept to a minimum during the meeting, both to ensure people with visual impairment were included and to ensure people joining on a mobile device (rather than a laptop or computer) could follow what was going on. Geographical-related factors, including language and time zones, also needed to be considered. Interpreters were required and the timing of the events was designed to suit participants in the different countries. Breakout room functions were used to allow nuanced discussion of the findings, before coming back to plenary to compare and contrast reflections. The breakout groups were organized around nationality to allow discussion to flow more freely in terms of language. Participants reported enjoying meeting fellow participants and hearing the findings. These events provided validation of the analysis and presented an opportunity to further elaborate on how the data were being interpreted.

Building on the initial themes, further analysis focusing on spatial description within the interviews was undertaken, which took into account contextual factors to explore spatial patterns of social processes affecting the behavior, choices, experiences, and perspectives of the participants (Rucks-Ahidiana & Bierbaum, 2015). Comparison of these spatial patterns was made between the data from the first and second interviews, validating the themes and suggesting data saturation. In addition, new themes and changes since the first round were noted.

## Results

The results found both “subjective” themes relating to feelings and “concrete” themes relating to material impacts affected the participants from across the five countries. The subjective themes included feelings of fear, shock, loss, destabilization, disorientation and uncertainty, as well as hope. The concrete themes related to concerns about health, finances, food security, vulnerability, and dependence. Preexisting disadvantage, discrimination, and marginalization relating to impairment, gender, and poverty were found to have been exacerbated by the COVID-19 crisis. These general themes are explored in more detail elsewhere (Wickenden et al., 2021). The results here reflect the spatial analysis and explore how the participants experienced space and culture during the pandemic. The emerging themes relating to these perspectives were found to fall into the broad categories of private and public space, as discussed below.

### Participants

In total, 75 participants were interviewed. Of these, 30 were from Bangladesh, 15 from Nepal, 10 from Kenya, 10 from Nigeria, and 10 from Uganda. More participants were from Bangladesh as participants there were drawn from two Inclusive Futures programs, while participants were only drawn from one program in the other countries. A gender balance was achieved with 38 males and 37 females participating. In terms of geography, 38 participants were from urban areas, 27 from rural areas, and 10 from peri-urban areas. Participants had a range of impairments: 10 had a physical impairment, seven had visual impairments, three had hearing impairments, 16 had intellectual impairments, six had psychosocial impairments, 10 had other impairments, two participants did not have impairments themselves but were parents of children (under 18) with multiple or complex impairments (one with severe autism and one with deafblindness), and 21 had multiple impairments. Participants had a range of ages, with 34 of them being under 29 years old, 38 being 30 to 49 years old, and three over 50 years old.

The vast majority of participants (70) had some form of education (apart from five participants who had none). Twelve participants had completed primary education, 12 had completed secondary education, and 41 had completed tertiary-level education. Five participants described their education as “other,” which included religious-based education as well as more vocational education pathways. The relatively high level of participant education is explained by the participant recruitment, as 40 of the participants were involved in a disability-inclusive employment program called Inclusion Works that forms part of Inclusive Futures, which skewed the sample toward more educated participants. With regard to work, 26 participants were employed or working, whereas 25 were unemployed. The remaining 24 participants were either students or described their occupation as “other.” Forty-nine participants described themselves as single, 24 participants were married, and two participants described their marital status as “other.” In terms of living arrangements, 42 participants had others depending on them, 11 participants depended on others, and 22 participants did not rely on others, nor did others rely on them. Of the 75 participants, 68 were interviewed twice. A total of 143 interviews were undertaken. The remaining seven participants declined a second interview or were unavailable.

### The Impact of the COVID-19 Pandemic on Experiences of Public Space

The way that the participants experienced, moved within, and occupied public space was found to be contextual in some instances, but universal in others. Some themes emerged that were relevant across all five countries and cultures, while other themes only emerged from certain countries and cultures. Specific locations considered to be public space that were referred to in the interviews included the neighborhood, offices, places of worship, markets and shops, schools, and health facilities. The first round of interviews was heavily weighted with negative experiences of public space by people with disabilities with reports of blatant discrimination and heightened exclusion. By the time the second round of interviews was complete, the picture was in general more positive. Many national lockdowns had been lifted and public life was slowly getting back to a situation more closely aligned with life prior to COVID-19. However, in some contexts, COVID-19 cases were beginning to rise, and further restrictions were either being introduced or threatened.

#### Discrimination Against People With Disabilities

A universal theme that emerged from all five countries was that during lockdowns, due to a perception that people with disabilities in general were more likely to be infected with COVID-19, they became ostracized. This perception was explained by preconceptions about people with disabilities being intrinsically vulnerable to infection. People with visual impairments relying on touch to navigate or people with physical impairments requiring assistance from others influenced how the public perceived all people with disabilities’ risk of infection. These perceptions turned into prejudice as anyone with disabilities became viewed as potentially infected. This had an impact on how they were treated by others in public spaces. In many contexts, preexisting exclusionary behavior became entrenched, with people with disabilities experiencing discrimination as they went about their business. As a man with albinism from Uganda reported,

Persons with disabilities have suffered twice; One for their impairment but also that they are easily suspected to be victims of covid 19 [infection] because everyone says we are vulnerable.

Some of this discrimination was manifested by increased mocking, teasing, and humiliation of people with disabilities. This seems to be particularly the case in Bangladesh but was also mentioned in other contexts. As one male participant from Bangladesh with multiple impairments commented,

Some people thought that if they saw any people with disabilities anywhere that their day is ruined. The community members still do mocking, teasing to the people with disabilities which is very alarming and uncomfortable.

These preconceptions made public life very challenging for some people with disabilities. Government guidance on social distancing was taken to the extreme in many contexts and used to justify the social exclusion of people with disabilities. Moving through public space since the COVID-19 crisis began appears particularly challenging for people with visual impairment. As one man who is deafblind in Bangladesh reflected,

Most of the person in my community thought that I carried the coronavirus and tried keep distance from me and did not talk with me.

A different male participant with visual impairment in Bangladesh recalled,

I have to touch everything to identify and this is not acceptable in the society. Everyone fears that maybe I am contagious.

#### Challenges Specific to People With Disabilities

Other groups of people with disabilities also reported specific challenges relating to COVID-19 restrictions and related economic impacts that affected their lives during the crisis. For example, people with albinism reported struggling to get the essential sun cream they need to protect their skin, which meant they could not go out in public. Also, people with physical disabilities reported challenges relating to wheelchair and other assistive device maintenance, which limited their mobility outside of their homes. People with hearing impairments reported difficulties in obtaining accessible information relating to COVID-19 and restrictions, meaning they were unsure of what was expected from them in public spaces.

#### Impact on Work

Another major challenge reported by participants across all five countries was that national lockdowns brought in to stop the spread of COVID-19 meant that they could not go to work. Although some participants had jobs that did not require them to occupy public spaces, the livelihoods of many across the five countries relied on trade with the general public or the provision of services to nonfamily members. Being restricted from accessing the spaces where they earned a living had an initial impact on their financial situation, but also secondary impacts on food security, housing, access to education and health services, and many other aspects of everyday life. The inability to work resulted in much stress and concern. As a female participant from Bangladesh with physical impairments stated,

At the March 25, 2020, Bangladesh Government declared the lock down which creates a thunderstorm on my head as I am totally dependent on my small shop. I did not have any other income source.

The second round of narratives indicated much relief when national lockdowns were lifted and people could return to their places of work. Although for some people, the lockdowns had changed their situation permanently. Some participants had lost their jobs completely. Others had their wages reduced or withheld on returning. This was a common situation for many people, but there was some suggestion that people with disabilities were at a particular disadvantage as it was more difficult for them to get a job in the first place due to various barriers and challenges. Once a job is lost, it is even harder to get a new one or return to the previous one. There were reports from Kenya of some people who had lost their jobs in urban areas returning to their ancestral villages. Despite the lockdown being lifted, they had yet to return.

#### Transport Challenges

Transport challenges around access to other public spaces was a universal theme across countries, with the cost of moving around becoming more expensive and being perceived as riskier (in terms of risk of infection) since the crisis had started. In Uganda in particular, transport costs have increased exponentially, creating a barrier to traveling, even after the lockdown was lifted. In several countries, some forms of public transport have had to reduce their capacity, to allow for social distancing, which has resulted in them avoiding picking up some people with physical disabilities due to the extra space needed for wheelchairs or other assistive devices. People with visual impairments reported relying on touch more than others when using public transport, which may increase their risk of infection. Some of the participants with physical disabilities reported requiring assistance to access public transport, which increases the risk of infection for them. Due to the perception that all people with disabilities are more likely to have COVID-19, public transportation became a space for entrenched discrimination. Some public transport operators ignored people with disabilities or refused to pick them up. Other people with disabilities who managed to enter the transport experienced negative interactions with other members of the public. One male participant from Bangladesh with visual impairments stated,

When visited to anyplace I used generally CNG [rickshaw] and sometimes by mistakenly I touched someone due to my impairment and most of them scold me like I am the carrier of the corona.

#### Interactions With the Authorities

Another emerging theme universal across the five countries was negative interactions between the participants with disabilities and the authorities, and in particular the police, in public spaces. The police are empowered by most states to ensure the safety, health, and possessions of people, yet our data suggest that the police contributed to making public spaces disabling during COVID-19 through their treatment of people with a range of impairments. Experiences range from the police not being very helpful (for example not assisting a person in Bangladesh with visual impairments to cross the road) to unnecessary and unjustified detention and the threat of violence experienced by someone with deafblindness in Nepal. The treatment of people with disabilities by the police during the crisis resulted in feelings of mistrust of the authorities. Participants felt fear and worry about future interactions with the police when outside their homes. One participant who was a woman from Nigeria with physical impairments recalled that when they asked the police for help navigating public space, this was the response:

They snubbed me and went on their way. They even threatened to arrest me despite my disability. So generally, the treatment was harsh.

There was particular concern about the police treatment of people with disabilities in Uganda, with narrations detailing accounts of the police shooting at or near people and using their guns to control crowds. One participant discussed a person with hearing impairment who had been shot in the leg by police for failing to obey an order. The victim was not trying to dissent, but had failed to act, as they had not heard the instruction. This worrying incident was also reported by journalists in Uganda in April 2020 (IBAHRI, 2020).

#### Impact on Education

Another major theme that came up across all five countries with regard to public space was the impact on educational facilities. As all participants were adults, the responses were either in relation to adult education they were enrolled in or in relation to education of either their own children or relatives or neighbors. Research involving the participation of children with disabilities themselves is needed to further understand the situation.

The closing of educational spaces brought about feelings of worry, desperation, and disappointment for many participants and their children. While education was disrupted across all five countries, the situation in Bangladesh appeared especially worrying. This was particularly the case for girls, who were reported as the most disadvantaged. With their education stopped, many families decided to arrange their marriages rather than wait for schools to reopen. Although this point is about girls in general, rather than just girls with disabilities, this is a concerning finding. As a man with multiple impairments from Bangladesh stated,

Many parents forced their girls for child marriage.

Despite reports of some learning moving to the online space, some people with disabilities or their children could not access it due to the lack of technology or internet, as was reported in Nepal by a male participant with deafblindness:

We don’t have online system (internet) so not being able to connect her to school. Other kids were able to join.

These online learning spaces were seen as a positive by some participants, as they opened up new pathways to education by overcoming barriers to access that existed in physical educational spaces.

### The Impact of the COVID-19 Pandemic on Experiences of Private Spaces

People with disabilities’ experience of private spaces was dominated by the impact of lockdowns and fear of catching COVID-19, which left them stuck in their homes, without visitors. For some, this feeling of being stuck was so strong that they felt like they were in prison or a cage. For example, a man with multiple impairments in Bangladesh said,

I felt like I was stuck in a prison because I did not have the habit to stay long time at home without any work.

#### Gender and Culture

In some countries, especially Bangladesh, women were noted to be especially confined within their homes, including because of cultural expectations. This made it harder for families without an adult male who could go out to buy necessities for them. Being restricted to crowded homes also meant that it was difficult to socially isolate if someone caught COVID-19. One woman with intellectual disabilities in Kenya also noted that they were forced to stay home, even as others started to leave their houses to get on with their lives. Here the conflict between the freedom to move around and stay safe was evident.

#### Boredom and Mental Health

Across all countries, one of the main feelings people with disabilities reported was being very bored at home, with nothing to do. In addition, many felt very lonely, and as people could no longer visit, some also felt abandoned. A woman with multiple disabilities in Bangladesh said,

Everyone in my community kept social distance which created an extra mental pressure on my mind like me and my family were the most abandoned one.

The lack of social contact and boredom negatively affected people with disabilities’ mental health and many felt unhappy. Children with intellectual disabilities especially struggled with the restrictions as they often did not understand why they were being kept home and this resulted in some behavioral problems that families struggled to cope with.

#### Access to Information

Being at home also limited people with disabilities information during the pandemic. One woman with deafblindness in Nepal who could no longer go to their association noted that

I am limited to home as a donkey because I do not get to speak to anyone, I have to stay on my own and I won’t know anything.

#### Stress and Rising Tensions

Being stuck at home increased tensions within families, especially when combined with increased financial problems because of lost livelihoods. A major struggle for people with disabilities during this time was the lack of food at home. Participants across all countries noted that men generally were stressed and frustrated by the situation and stated their perceptions that this was related to a marked increase in cases of domestic violence, although no domestic violence was directly reported by participants themselves. The participants did, however, detail increased tensions and arguments within their homes. For example, a woman with deafblindness in Bangladesh mentioned,

Since the outbreak of the epidemic, our family has had some quarrels at home.

Some of these tensions were directly related to concerns about the virus. In Uganda, for example, a woman with physical disabilities who had returned from her closed university to her home also noted that family members were isolating her within her own home as she was perceived as a threat.

During the second round of interviews as restrictions eased and people were able to work again, participants noted that there was more peace at home due to greater financial security.

In Kenya, one participant reported that there had been stories on increased sexual abuse of girls with disabilities due to them being stuck at home during the lockdown.

#### Social Interactions

People with disabilities in a number of countries noted that no longer being able to visit each other due to restrictions and fear of catching the virus negatively affected the quality of their relationships with their wider family and neighbors as they were no longer in regular communication with one another. For example, a man with multiple impairments from Bangladesh noted,

Due the security concern, I did not go to any of my relative house during the full lock down of COVID-19. Due to the lack of communication with the relatives there is gap of sincerity between us.

While in Nigeria, a man with albinism said,

The prevalent lockdown in these major states, especially in Ogun and Lagos States affected the cordiality between families and relations; not being able to cool down and pay courtesy visits to family and people, and gather together with loved ones.

In Nepal, a man with deafblindness had asked people to wear masks and people chose not to visit his home as a result, whereas in Bangladesh, a woman with physical impairments and her family were teased for taking precautions.

#### Disruption to Support Structures

Families struggled to support each other in the same way they had prior to the pandemic. However, in some cases, a wider family was what helped them to survive when they were sick with COVID and could not get food for themselves.

People with disabilities who were reliant on external caregivers struggled as caregivers or neighbors who normally assisted were unable to come and visit them due to lockdowns and social distancing. The long time spent at home increased some participant’s health problems. In addition, people whose assistive devices broke during this time were unable to get them fixed. All this, as well as the difficulties earning a livelihood, contributed to some people with disabilities feeling hopeless and dependent on others in a way that they had not been prior to the pandemic. For example, a woman with multiple disabilities in Bangladesh noted,

During the lock down, I was not able to step out from home as I need assistance to move anywhere. The neighbour who supposed to assist me to move anywhere also kept distance to the corona. I actually faced the bitterness of the world and I am totally helpless in that time.

A woman with physical disabilities in Nigeria whose calipers got damaged also noted,

This led to my dependence on others to get little things done, which is something I totally dislike. It was quite a frustrating experience. I felt like I was put in a cage, and always needed to call on passers-by to help me out with everything.

However, the experience of being stuck at home was not uniformly negative and one woman with visual impairments from Nigeria saw some positives to being stuck inside and the move online as it was an opportunity for personal development through the online opportunities now available.

## Discussion

The results give an insight into how people with disabilities in Bangladesh, Kenya, Nepal, Nigeria, and Uganda experienced both public and private spaces during the COVID-19 pandemic. Some experiences were universally reported across impairment groupings and countries. For example, using transport to move around in public became more challenging for people in all five countries due to increased costs, discrimination, and access issues, increasing the level of isolation felt by many. Other experiences were shaped by participants’ impairments, cultural contexts, gender, or an intersection of these.

For example, while the impact of the pandemic on educational spaces in general across countries and contexts has been widely reported (UNICEF, 2021), the impact in Bangladesh was found to be particularly worrying. People with disabilities were in many cases already being left behind from education and the COVID-19 crisis has resulted in them falling even further behind. As the United Nations Partnership on the Rights of Persons with Disabilities (UNPRPD, 2021) reported, “The COVID-19 pandemic exposed the shortcomings, fragilities, risks and inequalities in the education of learners with disabilities within and across countries” (p. 4). Our findings suggest in education spaces, discrimination based on disability is intersecting with inequalities linked to social norms relating to the education of girls in Bangladesh specifically. The impact of gendered social norms in this context has been shown to have a significant impact on the education of girls (Aregu et al., 2018).

The findings provide useful insights into experiences of police brutality in public spaces. Such abuses of power from people in positions of authority have been shown to affect many people and communities around the world. Some governments and police departments have been accused of using the pandemic as a pretext for abuse (Amnesty International, 2020). Our research indicates that people with disabilities may face specific discrimination in public spaces during the pandemic, resulting in abuse and unfair treatment by people in positions of authority.

Across all five countries, people with disabilities reported being ostracized in public spaces due to assumptions that they would have or spread COVID-19. This finding resonates strongly with discussions around the relationship between vulnerability and disability. For example, writing on this topic Sparf (2016) warns of the dangers of assuming someone is vulnerable without considering their individual circumstances that shape how they experience environments. Scully (2013) critically assesses underlying assumptions that people with disabilities have specific vulnerabilities, distinct from the ontological vulnerability of all human life. She argues that vulnerability is ultimately established through social and political decisions. The COVID-19 crisis has highlighted universal human vulnerability in an unprecedented way. Although it could be argued that certain impairments may increase risk of infection (for example, people with visual impairments may be at increased risk due to their potential reliance on touch), assumptions that all people with disabilities are automatically more vulnerable and likely to be spreaders/carriers of COVID-19 can result in discrimination and exclusion.

The debate around disability and vulnerability must not result in this group being excluded from the policy sphere. The WHO (2020), along with other international bodies, was very clear with their warning that emergency measures must not discriminate on the basis of disability. While disability is not always linked to vulnerability, if groups such as people with disabilities are not specifically included in policy and planning, then vulnerability may actually be produced and risk to those individuals increased. Evidence from South Africa has shown that the COVID vaccine is more urgently needed by some people with disabilities, due to underlying health conditions and/or preexisting social vulnerability associated with poverty and stigma. If the particular circumstances and specific assistance required by persons with disabilities are overlooked by policymakers, then this group faces a potentially disproportionate disadvantage due to the pandemic (Hart et al., 2021).

The COVID-19 pandemic triggered the worst global job crisis in decades (International Labour Organization [ILO], 2020). In pre-COVID times, the exclusion experienced by people with disabilities is well documented, as they regularly face a multitude of social, cultural, and access barriers to work (Wickenden et al., 2020). Our data showed that as lockdowns were eased and economies started to open back up, the exclusionary practices of old returned and, in some instances, were even more severe. The pandemic had made the bleak work situation even worse, with people with disabilities facing more disadvantage when trying to secure full and productive employment and decent work. The pandemic presented a chance for employers to try and do things differently as economies re-started, such as adapting more flexible work patterns, allowing home working, and investing in assistive technology. Although many of these initiatives would primarily benefit those working in offices, our data suggest that a broader opportunity for employers to become more inclusive was missed, and many workspaces are now more disability exclusive than in pre-COVID times.

With regard to private spaces, the fear of COVID-19 left many people with disabilities concerned and worried. The national lockdowns resulted in feelings of isolation and desperation. Boredom was reported across the different countries and cultures, as the crisis had a negative impact on people’s mental health. People with disabilities feeling they are being segregated and excluded from the community have been documented in pre-COVID-19 times (Merrells et al., 2019). Our research suggests that the pandemic has exacerbated this situation. People with intellectual disabilities were found to be particularly negatively affected, as many struggled to understand the restrictions and why they were being imposed.

The lockdowns also had a major impact on the support networks of the participants, including having less support from families and neighbors. Literature suggests that social interactions within environments have the potential to deliver positive effects on both mental health and well-being, but that people with disabilities often have fewer opportunities to engage (Tough et al., 2017). Our data showed clearly that support networks were badly disrupted for people with disabilities during COVID-19, resulting in challenges to everyday life and the occupation of private space. Lockdown situations resulted in increased tensions between families, with many arguments being linked to financial stresses.

The wider significance of our findings is that people with disabilities must not be left behind during emergencies. To ensure this does not happen, people with disabilities and the organizations that represent them must be involved in and contribute to disaster planning undertaken in all countries and contexts. Only when full and meaningful participation in society across all spaces and cultures is achieved can be ensured no one is left behind.

### Limitations

There were some limitations to this study that should be considered when interpreting the results. First, despite aiming for consistency in the research methodology across the five countries, the time between interview rounds varied. For some participants, the gap was shorter than others due to logistical challenges. Although changes over time were identified and were found to be heavily influenced by specific country lockdown circumstances, greater consistency with regard to the gap between rounds would have been preferred. In general, interviewees were found to be more open in the second round. It is possible that those who only had a short gap may have been less forthcoming about their experiences, which influenced the data and analysis.

This study did not include people with disabilities who are in institutional care. Further research involving this group may provide further insights into very specific spatiocultural experiences during the pandemic. Another limitation relates to marginality. Despite deliberately recruiting participants from commonly excluded groups (e.g., people with deaf-blindness, intellectual, and psychosocial disabilities), other aspects of the participants’ identities suggest that the participants were not necessarily among the most marginalized within society. For example, the majority of the participants were educated, and many were employed. Further research involving the most marginalized members of society who may experience multiple or intersecting inequalities may provide different results.

Children with disabilities did not participate in this study, but certain topics relevant to them (such as education) are discussed. Further research involving the participation of children with disabilities themselves is needed to better understand the situation.

Despite aiming for to deliver an inclusive and accessible research process, it is possible that the online nature of the study may have been unsatisfactory and limiting for some people in terms of both access to technology and communication. This may have further impacted the sample of people who were interviewed, with those without access or challenges around communication opting not to participate.

## Conclusion

The COVID-19 crisis has resulted in an unprecedented disruption to everyday life across cultures, countries, and contexts. Our research shows that the pandemic has exacerbated existing marginalization for people with disabilities in the low- and middle-income contexts of Nigeria, Bangladesh, Nepal, Kenya, and Uganda. Data from 143 narrative interviews with participants who had a range of impairments were, first, analyzed thematically and, second, from a spatial perspective, allowing us to explore patterns of social processes, behaviors, and experiences across the five countries. Themes were both concrete and subjective, often with the two being interconnected. The participants chose to discuss how these impacts affected both their occupation of private and public space.

Some themes were found to be universal across countries and contexts. National lockdowns resulted in feelings of boredom, frustration, and loneliness being experienced as people had to isolate. Material impacts such as having to stay at home rather than work resulted in exacerbated poverty, food insecurity, and concern over financial stability. As public life was shut down, usual support networks were halted, causing significant disruption to the lives of people with disabilities, including lack of access to the services they usually used, such as health care and rehabilitation. In addition, family relations were put under increased pressure.

Some impacts of the pandemic were found to be culturally specific. Social and cultural norms in Bangladesh resulted in women being more affected than men by some aspects of the crisis (for example, in accessing education and being able to leave the house). Unjust treatment of people with disabilities by the police was found to be an issue in all five countries, but in Uganda, in particular, this was a concern about the level of police violence and the lack of accessible communication available.

Across all five countries, people with disabilities were perceived by the general public as being more at risk of being infected and contagious. This resulted in increased exclusion in public places and being ostracized. These damaging misconceptions resonate with how impairment and vulnerability are often intrinsically linked with people with disabilities. Often, people with disabilities are regarded as a homogeneous group, with individual characteristics, contexts, and concerns being disregarded.

Although this research has contributed to understanding about the impact on how people with disabilities experienced and occupied space during the pandemic, further research could investigate culturally and contextually appropriate pathways to address exacerbated marginalization in the five countries. In general, to overcome the challenges caused by COVID-19 and future crises, national and societal responses must be fully disability inclusive, with people with disabilities themselves being consulted on both the process and the practice.
